# Computational and Experimental Analyses for Pathogenicity Prediction of *ACVRL1* Missense Variants in Hereditary Hemorrhagic Telangiectasia

**DOI:** 10.3390/jcm12155002

**Published:** 2023-07-29

**Authors:** Toru Iwasa, Akihiro Urasaki, Yuki Kakihana, Nami Nagata-Akaho, Yukihiro Harada, Soichi Takeda, Teruhisa Kawamura, Isao Shiraishi, Kenichi Kurosaki, Hiroko Morisaki, Osamu Yamada, Osamu Nakagawa

**Affiliations:** 1 Department of Molecular Physiology, National Cerebral and Cardiovascular Center Research Institute, 6-1 Kishibe-Shimmachi, Suita, Osaka 564-8565, Japan; tiwasa@ncvc.go.jp (T.I.);; 2 Department of Pediatric Cardiology, National Cerebral and Cardiovascular Center, 6-1 Kishibe-Shimmachi, Suita, Osaka 564-8565, Japan; 3 Laboratory of Stem Cell and Regenerative Medicine, Department of Biomedical Sciences, College of Life Sciences, Ritsumeikan University, 1-1-1 Noji-Higashi, Kusatsu, Shiga 525-8577, Japan; 4 Department of Advanced Medical Technologies, National Cerebral and Cardiovascular Center Research Institute, 6-1 Kishibe-Shimmachi, Suita, Osaka 564-8565, Japan; 5 Department of Medical Genetics, Sakakibara Heart Institute, 3-16-1 Asahi-cho, Fuchu, Tokyo 183-0003, Japan

**Keywords:** hereditary hemorrhagic telangiectasia, ACVRL1, ALK1, BMP, SMAD signaling

## Abstract

Hereditary hemorrhagic telangiectasia (HHT) is a vascular disease caused by the defects of ALK1/ACVRL1 receptor signaling. In this study, we evaluated 25 recently identified ACVRL1 missense variants using multiple computational pathogenicity classifiers and experimentally characterized their signal transduction capacity. Three extracellular residue variants showed no detectable cell surface expression and impairment of bone morphogenetic protein 9 (BMP9) responsiveness of SMAD-dependent transcription in luciferase assays. Four variants with amino acid replacement in the motifs essential for the intracellular kinase function lost SMAD-dependent signaling. Most of other variations in the kinase domain also caused marked downregulation of signaling; however, two variants behaved as the wild-type ACVRL1 did, while computational classifiers predicted their functional abnormalities. Three-dimensional structure prediction using the ColabFold program supported the significance of the L45 loop and NANDOR domain of ACVRL1 for its association with SMAD1 and BMPR2, respectively, and the variations in these motifs resulted in the reduction of SMAD signaling. On the other hand, two of the GS domain variants maintained high signal transduction capacity, which did not accord with their computational pathogenicity prediction. These results affirm the requirement of a combinatory approach using computational and experimental analyses to accurately predict the pathogenicity of ACVRL1 missense variants in the HHT patients.

## 1. Introduction

Hereditary hemorrhagic telangiectasia (HHT), or Osler–Weber–Rendu disease, is a vascular disorder of autosomal dominant inheritance. The Curaçao criteria is widely used for the diagnosis based on its features: spontaneous and recurrent epistaxis; mucocutaneus telangiectasia; visceral lesions, such as gastrointestinal telangiectasia and pulmonary/hepatic/cerebral/spinal arteriovenous malformations; and family history [1]. A definitive diagnosis of HHT is made if three or more criteria are satisfied. Defects in vascular ALK1 signaling are implicated in its pathogenesis, and heterozygous variations of the genes encoding endoglin (*ENG*), activin A receptor like type 1 (*ACVRL1*), SMAD family member 4 (*SMAD4*) and bone morphogenetic protein 9 (*GDF2*) have been identified to be causative in HHT [2].

The ACVRL1 receptor, also called ALK1, forms a complex with bone morphogenetic protein (BMP) receptor type 2 (BMPR2) and endoglin, which is activated upon the BMP9/BMP10 ligand binding and provokes SMAD-dependent transcriptional regulation of downstream genes [3,4]. ALK1 signaling is essential for normal vascular formation during embryonic development, and the mice and zebrafish null for *Acvrl1*/*acvrl1* show embryonic lethality due to severe vascular abnormalities [5,6]. Variations in the causative genes were found in most of the patients with a definite HHT diagnosis, and approximately half of them are in *ACVRL1*. The haploinsufficiency of *ACVRL1* and other disease genes is accepted as a pathogenic mechanism of HHT while protein products of particular missense variants may remain and show diverse modes of functional deficits in patient cells.

The American College of Medical Genetics and Genomics (ACMG) standards and guidelines have been used to evaluate the clinical significance of novel variants [7]. The ACMG guideline includes the utilization of in silico pathogenicity prediction programs. The computational pathogenicity classification is, however, not regarded as decisive (evidence of pathogenicity is supportive: PP3) in the guideline, and the results are sometimes inconsistent among different programs. The experimental studies of gene product functions can provide stronger evidence, PS3, to support the presence of a damaging effect by a certain missense variation. Since previous studies reported functional analyses of ACVRL1 variant proteins [8,9,10], numerous different missense variations have been published or registered in the public databases, such as those by the Associated Regional and University Pathologists (ARUP) laboratories and ClinVar archive, which are present in the *ACVRL1* exons that encode the extracellular region, intracellular kinase domain and functional motifs conserved among ALK family receptors.

In the present study, we examined recently identified *ACVRL1* missense variants using multiple computational pathogenicity classifiers and characterized their signal transduction capacity in cell culture-based experiments. Our results showed that the evaluation with computational and experimental analyses was not consistent in several variants, supporting a notion that a combinatory approach was necessary to accurately estimate the pathogenicity of *ACVRL1* missense variations detected in the HHT patients.

## 2. Materials and Methods

### 2.1. Patient and Computational Pathogenicity Prediction

To obtain the information of *ACVRL1* missense variants found in the HHT patients, we utilized the original papers and reviews, public databases and unpublished clinical cases genetically analyzed at our institute. Variant detection of the patients listed in Table 1 was performed with Sanger sequencing of the coding regions and the exon–intron boundaries of *ACVRL1* and *ENG*.

Four web-based programs were used for the computational pathogenicity prediction: SIFT (http://sift.jcvi.org/, accessed on 23 November 2022), PROVEAN [https://www.jcvi.org/research/provean/, accessed on 23 November 2022 via Varsome (https://varsome.com/)], PolyPhen-2 (http://genetics.bwh.harvard.edu/pph2/, accessed on 23 November 2022) and PANTHER (http://www.pantherdb.org/, accessed on 20 November 2022) [11,12,13,14].

### 2.2. ACVRL1 and Endoglin Expression Plasmids

The expression plasmid constructs for wild-type human ACVRL1 and its variants were prepared with site-directed mutagenesis PCR of the pcDNA3-HASL-ALK1(WT) plasmid provided by Drs. Kohei Miyazono and Tetsuro Watabe [15,16]. Inverse PCR method was employed using the PrimeSTAR MAX enzyme (Takara Bio, Kusatsu, Japan) and oligonucleotide primers with designed mutations. The expression plasmid for human endoglin, pDEF3-Endoglin, was provided by Dr. Fumiko Itoh [17]. HA-tag and FLAG-tag at the C-terminus were used for ACVRL1 and endoglin plasmids, respectively.

**Table 1:** Clinical features of unpublished HHT cases with ACVRL1 missense variations.

Variant	Age, Sex	Epistaxis	TE	PVM	HVM	BVM	Family History	Curaçao Criteria
D176Y	67, M	+	+	−	−	−	+	Definite
D235Y	67, F	+	+	−	+	−	+	Definite
D235Y	61, M	+	+	−	+	−	+	Definite
P424T	61, F	+	−	−	+	−	−	Definite
D437G	81, F	−	−	+	+	−	+	Definite
D437G	46, M	+	+	+	−	−	+	Definite
R479P	28, F	+	+	−	−	−	+	Definite
R484L	49, F	+	−	−	+	−	+	Definite

TE, telangiectasia; PVM, pulmonary vascular malformations; HVM, hepatic vascular malformations; BVM, brain vascular malformations; M, male; F, female. Two patients with a D235Y variation were from unrelated families. Two patients with a D437G variation belonged to the same family.

### 2.3. Cell Culture and Plasmid Transfection

NIH-3T3 fibroblast cell line was maintained using Dulbecco’s Modified Eagle’s Medium (DMEM) (Nacalai Tesque, Kyoto, Japan) supplemented with 10% fetal bovine serum (Gibco, Thermo Fischer Scientific, Waltham, MA, USA) and Penicillin (100 units/mL)/Streptomycin (100 μg/mL) (FUJIFILM Wako Pure Chemical, Osaka, Japan). Plasmid transfection was performed in Opti-MEM (Invitrogen, Thermo Fischer Scientific, Waltham, MA, USA) using Lipofectamine 2000 (Invitrogen).

### 2.4. Immunocytochemistry

NIH-3T3 cells were transfected with wild-type or variant ACVRL1 plasmid (400 ng per a 35-mm glass bottom dish). Immunocytochemistry was performed using anti-human ALK-1 antibody AF370 (R&D systems, Bio-Techne, Minneapolis, MN, USA), anti-Goat IgG Alexa Fluor 488 (Invitrogen) and 4′,6-diamidino-2-phenylindole (DAPI) (Invitrogen). Fluorescent images were taken using FV3000 confocal microscope (Evident, Tokyo, Japan).

### 2.5. Luciferase Reporter Assay

Three luciferase reporter constructs, pGL4-BRE-MLP-fLuc (BRE-luciferase) [15,16], pGL4-mId1-1.6Pr (ID1-luciferase) and pGL4-BMPR2(int)3WT-MLP-fLuc (BMPR2-luciferase) [16], were used to examine SMAD-dependent signal transduction. BRE-luciferase and BMPR2-luciferase plasmids were provided by Drs. Kohei Miyazono and Daizo Koinuma. ID1-luciferase plasmid was generated by the PCR amplification of 1.6-kilobase mouse *Id1* promoter [15] and insertion into the pGL4.10 [luc2] vector (Promega, Madison, WI, USA). After 1 h of pre-conditioning in DMEM containing 0.1% bovine serum albumin, NIH-3T3 cells were transfected for 4 h with 200 ng of luciferase reporter plasmid, 50 ng of cytomegalovirus promoter β-galactosidase reporter plasmid and 100 ng of ACVRL1 plasmid per a well of 24-well plates. Cells were then treated with BMP9 (6.25, 25 or 100 pg/mL) or vehicle for 24 h. To analyze the effect of the endoglin co-expression, cells were co-transfected with 200 ng of BRE-luciferase plasmid, 50 ng of cytomegalovirus promoter β-galactosidase plasmid, 0.5 ng of ACVRL1 plasmid and 5 ng of endoglin plasmid or vector, followed by the treatment with BMP9 (5 pg/mL) for 24 h. Luciferase and β-galactosidase activities were measured using FLUOStar Omega (BMG LABTECH, Ortenberg, Germany). Reproducibility of the results was confirmed in at least two independent experiments.

### 2.6. Western Blot Analysis

NIH-3T3 cells were transfected with wild-type or variant ACVRL1 plasmid (800 ng per a well of 12-well plates). Twenty-four hours after the transfection, cells were serum-starved for 4 h and treated with BMP9 (100 pg/mL) or vehicle for 20 min. Western blot analysis was performed using following antibodies: phosphorylated SMAD1/5/9 antibody #13820 (Cell Signaling Technology, Danvers, MA, USA), SMAD1 antibody #6944 (Cell Signaling Technology), Glyceraldehyde 3-phosphate dehydrogenase (GAPDH) antibody MAB374 (Millipore, Merck, Burlington, NJ, USA). SuperSignal West Femto Maximum Sensitivity Substrate (Thermo Fischer Scientific) or ECL^TM^ Prime Western blotting detection reagent (Merck, Darmstadt, Germany) was used for the detection.

### 2.7. Computational Structure Prediction

The three-dimensional structures of the ACVRL1-BMPR2-SMAD1 trimer as well as the ACVRL1-BMPR2 and ACVRL1-SMAD1 dimers were predicted by using ColabFold (AlphaFold2 using MMseqs2) (https://colab.research.google.com/github/sokrypton/ColabFold/blob/main/AlphaFold2.ipynb, accessed on 22 July 2022) [18]. The amino acid sequences of the receptor intracellular kinase domains, ACVRL1 [amino acid no. (aa) 195-503] and BMPR2 (aa 197-512), and the SMAD1 MH2 domain (aa 271-465) were analyzed with the default settings and heterooligomer option. Predicted local distance difference test (pLDDT) and predicted alignment error (pAE) graphs of five models were generated to select the one with the best prediction quality. The images of protein structures were generated using the PyMOL Molecular Graphics System (Schrödinger, New York, NY, USA).

### 2.8. Statistical Analysis

Tukey’s or Dunnett’s test was performed using the Prism 9 software (GraphPad Software, Boston, MA, USA). The method used in each experiment was described in the figure legends.

## 3. Results

### 3.1. ACVRL1 Variants in this Study and Profiles of Unpublished Clinical Cases

**Figure 1:**
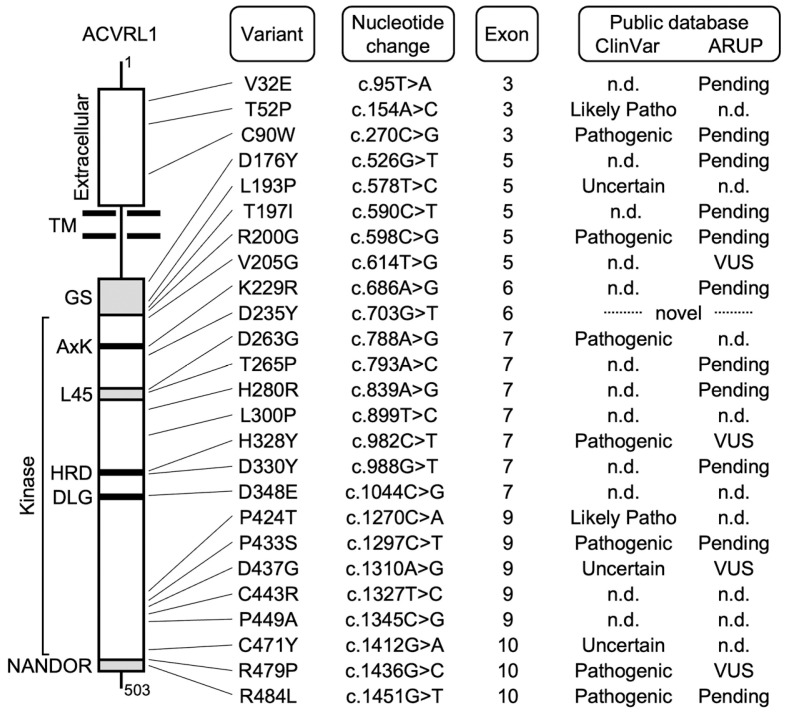
Characteristics of ACVRL1 missense variants selected for computational and experimental analyses. In the schematic structure of ACVRL1, the functional motifs/domains conserved among ALK family receptors are filled in grey, and those shared by protein kinases are filled in black. AxK, AxK motif; DLG, DLG motif; GS, GS domain; HRD, HRD domain; L45, L45 loop; Likely Patho, Likely pathogenic; NANDOR, NANDOR domain; n.d., not deposited; TM, transmembrane domain; Uncertain, Uncertain significance; VUS, variant of uncertain significance. References: V32E [19,20], T52P [21], C90W [20], D176Y [24], L193P [22], T197I [25], R200G [19,20], V205G [19,20], K229R [26], D263G [37], T265P [24], H280R [19,20,28,30], L300P [20], H328Y [19,20,27,29], D330Y [10,31,32], D348E [20], P424T [38], P433S [33,34], D437G [35], C443R [20], P449A [20], C471Y [20], R479P [28], R484L [36].

The present study focused on *ACVRL1* variants with a single amino acid substitution recently published or deposited in the ARUP and ClinVar databases [1,19,20,21,22,23] as well as those found in the genetic analyses performed at our institute. All the variants were reported for at least one case with a definite HHT diagnosis according to the Curaçao criteria or were described as being derived from the HHT patients in previous reports (Figure 1) [10,19,20,21,22,24,25,26,27,28,29,30,31,32,33,34,35,36,37,38]. These variations were not listed in the gnomAD databases (v2.1.1 and v3.1.2) and the 38KJPN database for the Japanese population [39,40].

Some of them were detected in unpublished clinical cases at our institute, including a novel variant, c.703G>T (D235Y) (Table 1). The first patient with a D235Y variation was a 67-year-old woman. She had presented recurrent epistaxis for approximately 10 years. To reduce nasal bleeding, the sphenopalatine artery ligation, internal maxillary artery embolization and nasal mucosa cauterization were performed when she was 65 and 67 years old. She had characteristic telangiectasias on the lip, tongue and fingers. Enhanced computed tomography revealed tortuous and dilated hepatic arteries and multiple hepatic arteriovenous shunts, but pulmonary and cerebral vascular malformations were not detected. Her father also presented recurrent epistaxis, while he did not have an opportunity of the evaluation for the HHT diagnosis. The second D235Y patient was a 61-year-old man. He experienced recurrent epistaxis and had telangiectasias on the fingers. Computed tomography demonstrated multiple arteriovenous shunts in the liver and pancreas but not in the lung. His uncle and aunt were clinically diagnosed as HHT. These patients satisfied the Curaçao criteria for the definite HHT diagnosis. They belonged to unrelated families but lived in the vicinity, and the genetic analysis at our institute was requested by the same hospital. In addition, the genetic analyses at our institute identified the patients with the D176Y, P424T, D437G, R479P or R484L variation, each of which was previously reported in only one case [24,28,35,36,38]. Clinical features of those patients were described in Table 1 and Supplemental Information.

### 3.2. Computational Pathogenicity Prediction of ACVRL1 Missense Variants

The ACVRL1/ALK1 receptor (NCBI Reference Sequence: NP_000011.2) is a single-pass membrane protein that consists of the extracellular (aa 22-118), transmembrane (aa 119-141) and intracellular (aa 142-503) regions. The intracellular region possesses a serine/threonine protein kinase domain (aa 193-493) and the motifs structurally conserved among ALK family receptors, such as the GS domain (aa 172-201), L45 loop (aa 262-270) and NANDOR domain (aa 479-489) [8]. We selected 25 recently identified *ACVRL1* missense variants in various regions and functional domains for the computational and experimental analyses (Figure 1).

We first performed computational pathogenicity prediction of these variants using four systems, PolyPhen-2, SIFT, PROVEAN and PANTHER, which mainly relied on evolutional conservation and structural/chemical properties of amino acid residues [11,13,41,42]. Being consistent with the fact that they were detected in the patients, many of these variants were predicted to have significant pathogenicity by all the programs. However, three variants (V32E, T52P and T265P) were given incongruous results and were categorized into non-pathogenic groups, such as “benign”, “tolerated” or “neutral”, by more than one classifier (Table 2).

### 3.3. Subcellular Localization and Signal Transduction Capacity of Extracellular Residue Variants

**Figure 2:**
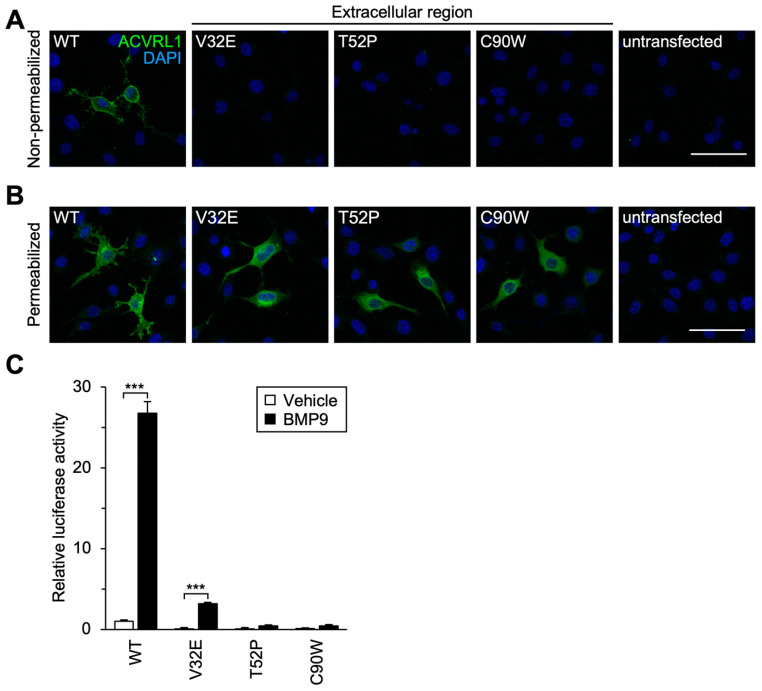
Subcellular localization and signal transduction capacity of extracellular residue variants. (**A**) The extracellular residue variants were not detected on the cell surface. Immunocytochemistry in non-permeabilized NIH-3T3 cells. Scale bar: 40 μm. (**B**) The extracellular residue variants were detected in the cytoplasm of permeabilized NIH-3T3 cells. Scale bar: 40 μm. (**C**) The extracellular residue variants showed marked down regulation of BMP9-induced signal transduction in BRE-luciferase assays. Results are expressed as fold induction over the value obtained for the cells expressing wild-type (WT) ACVRL1 with the vehicle treatment. *** *p* < 0.001 (Tukey’s test).

To examine how accurately these classifiers can predict functional alteration of ACVRL1 missense variant proteins, we examined their capacity to transduce intracellular signaling in cultured cells. According to the previous studies [8,9,43], we transfected mouse NIH-3T3 cells, which expressed a low level of endogenous Acvrl1 protein, with an expression plasmid for wild-type or variant ACVRL1. Immunocytochemistry using an antibody recognizing the extracellular region of ACVRL1 revealed that three extracellular residue variants (V32E, T52P and C90W) did not show detectable signals on the cell surface while wild-type ACVRL1 was localized on the cell surface (Figure 2A). Significant amounts of V32E, T52P and C90W variants were detected in the cytoplasm when the staining was performed after the membrane permeabilization with Triton X-100 (Figure 2B), suggesting that the mutation of V32, T52, or C90 residue affected the cell surface presentation of ACVRL1 receptor. We then studied the effects of these three variations upon BMP9-induced SMAD-mediated transcriptional activity. As expected, NIH-3T3 cells expressing one of these extracellular residue variants showed an apparent reduction in the BMP9-induced activation of BRE-luciferase, which was a well-established reporter for the SMAD-dependent transcriptional regulation (Figure 2C) [15]. Signal transduction mediated by the V32E variant was markedly downregulated but still remained significantly, suggesting that an undetectable amount of V32E receptor was presented on the cell surface. It is noteworthy that V32E and T52P variants were categorized into non-pathogenic groups by multiple computational classifiers (Table 2) in contrast to their abnormal signal transduction capacity in the experimental analyses.

### 3.4. Importance of Functional Motifs and Conserved Residues in Intracellular Kinase Domain

**Figure 3:**
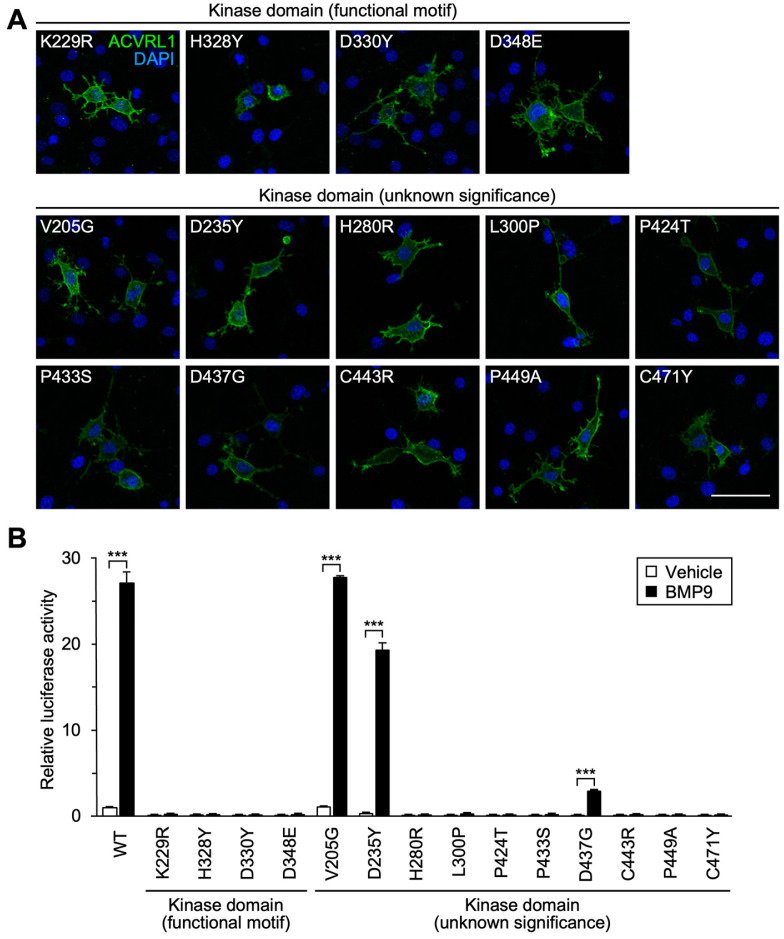
Subcellular localization and signal transduction capacity of kinase domain variants. (**A**) All kinase domain variants were detected on the cell surface. Immunocytochemistry in non-permeabilized NIH-3T3 cells. Scale bar: 40 μm. (**B**) Most of the kinase domain variants failed to mediate BMP9-induced transcriptional activation, while the D437G variant showed weak but statistically significant signal transduction in BRE-luciferase assays. In contrast, V205G and D235Y variants kept high BMP9-induced signaling activity comparable to that of wild-type ACVRL1. Results are expressed as fold induction over the value obtained for the cells expressing wild-type (WT) ACVRL1 with the vehicle treatment. The data for wild-type ACVRL1 are same as those in Figure 2C. *** *p* < 0.001 (Tukey’s test).

We next examined the function of 14 missense variants of the serine/threonine kinase domain that encompassed most of the ACVRL1 intracellular structure. ACVRL1 phosphorylates SMAD1/5/9 as a major downstream event of ACVRL1-BMPR2 receptor complex activation, and it contains several essential motifs for the kinase activity [44]. K229 and D348 are in the AxK and DLG motifs, respectively, which are essential for the interaction with ATP and magnesium ion, while the HRD motif (aa 328-330) is required for the association with the substrate serine residue [45]. Indeed, four variants with the alteration of conserved residues in those motifs, K229R, H328Y, D330Y and D348E, failed to mediate the BRE-luciferase activation in response to BMP9, although they were correctly expressed on the cell surface (Figure 3A,B). 

We also selected 10 missense variants that had an alteration of the amino acid residues highly conserved among species (Table 2) but not known to be indispensable for the kinase activity. All of them were localized on the cell surface (Figure 3A), and the cells expressing eight variant proteins showed the absence or marked reduction of downstream SMAD signaling (Figure 3B), which got along well with the importance of ACVRL1 kinase function. In contrast, V205G and D235Y variants behaved as the wild-type ACVRL1 did in the luciferase analysis (Figure 3B), although computational classifiers suggested their functional abnormalities (Table 2).

### 3.5. Structural Prediction of ACVRL1-SMAD1 Interaction and Impact of Missense Variations in L45 Loop upon Signal Transduction Activity

**Figure 4:**
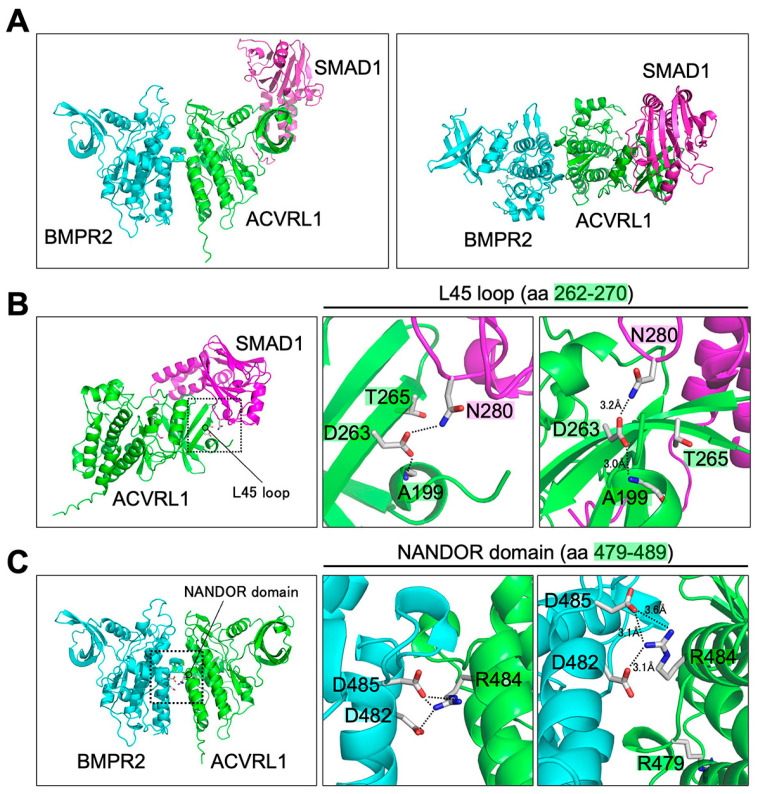
Three-dimensional structural prediction of ACVRL1 interaction with SMAD1 and BMPR2. ACVRL1 kinase domain (green; aa 195-503), BMPR2 kinase domain (cyan; aa 197-512) and SMAD1 MH2 domain (magenta; aa 271-465) were applied to the structural prediction using ColabFold. (**A**) The predicted structure of the ACVRL1-BMPR2-SMAD1 trimer viewed from two different directions. A top view of the left image is on the right. (**B**) ACVRL1-SMAD1 dimer structure (left) with an enlarged view of the dotted box (middle). The figure on the right shows the area in the middle figure viewed from another direction. Side chains of ACVRL1 D263 and T265, that of SMAD1 N280 and a main chain of ACVRL1 A199 are depicted. In the ACVRL1-SMAD1 interface shown on the right, the interaction between ACVRL1 D263 in the L45 loop and SMAD1 N280 was predicted. Intramolecular interaction between ACVRL1 D263 and A199 was also indicated. D263 and T265 of ACVRL1 are localized in the same β sheet. (**C**) ACVRL1-BMPR2 dimer structure (left) with an enlarged view of the dotted box (middle). The figure on the right depicts the image in the middle figure viewed from another direction. In the ACVRL1-BMPR2 interface shown on the right, the interaction between ACVRL1 R484 in the NANDOR domain with BMPR2 D482 and D485 was predicted. On the other hand, ACVRL1 R479 did not show direct interaction with BMPR2. Carbon, oxygen and nitrogen atoms are represented by gray, red and blue, respectively, and hydrogen atoms are omitted for clarity. Dotted lines indicate possible amino acid interaction with the distance less than 4 Å.

ACVRL1/ALK1 belongs to the ALK receptor family that is composed of ACVR1/ALK2, BMPR1A/ALK3, ACVR1B/ALK4, TGFBR1/ALK5, BMPR1B/ALK6 and ACVR1C/ALK7 [46]. Preceding investigations on different ALK family receptors indicated conserved functional domains that also existed in ACVRL1 [46]. For example, the L45 loop of TGFBR1/ALK5 was shown to physically associate with SMAD2 [47,48] while it was untested whether ACVRL1 also interacted with SMAD proteins via its L45 loop (aa 262-270). We then employed ColabFold, a newly-developed computational system based on the AlphaFold2 program [18], for a three-dimensional prediction of the interaction between ACVRL1 and SMAD1. ColabFold predicted stable trimer formation among the ACVRL1 kinase domain (aa 195-503), BMPR2 kinase domain (aa 197-512) and SMAD1 MH2 domain (aa 271-465) (Figure 4A). In addition to a sufficiently high pLDDT score and a sufficiently low pAE value at the contacts, the contact surfaces of the trimer are free of major collisions, and the electrostatic and shape complementarity makes it a very plausible complex structure. The analysis using the ACVRL1-SMAD1 dimer indicated their interaction via the L45 loop (Figure 4B). Especially, it was predicted that D263 of ACVRL1 was located in the β sheet at the ACVRL1-SMAD1 interphase and was in close contact with N280 of SMAD1 (Figure 4B).

**Figure 5:**
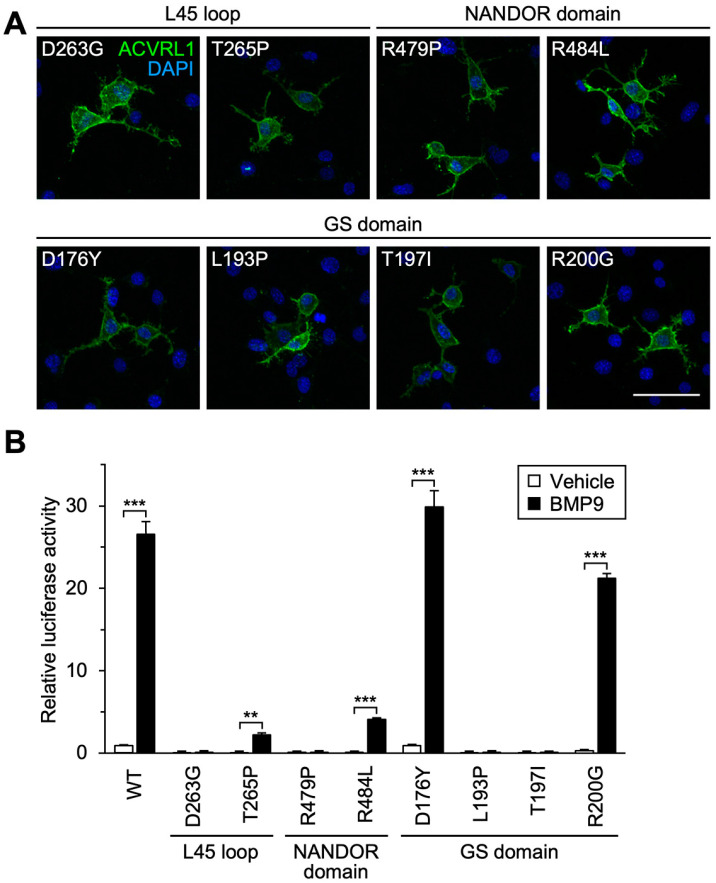
Influence of missense variations in functional domains conserved in ALK family receptors. (**A**) ACVRL1 receptors with a variation in the L45 loop, NANDOR domain or GS domain were localized on the cell surface. Immunocytochemistry in non-permeabilized NIH-3T3 cells. Scale bar: 40 μm. (**B**) Missense variations in those functional domains markedly down regulated BMP9-induced transcription in BRE-luciferase assays while that mediated by T265P and R484L variants still had statistical significance. In contrast, the cells expressing D176Y and R200G variants showed high transcriptional activation in response to BMP9. Results are expressed as fold induction over the value obtained for the cells expressing wild-type (WT) ACVRL1 with the vehicle treatment. The data for wild-type ACVRL1 are same as those in Figure 2C. ** *p* < 0.01; *** *p* < 0.001 (Tukey’s test).

Consistently, a D263G variation in the L45 loop resulted in the loss of BMP9-induced BRE-luciferase reporter activity while the variant protein was properly expressed on the cell surface (Figure 5A,B). We also tested the effect of the T265P variation, which resulted in an amino acid replacement in the same β sheet of the L45 loop. Increase in the BRE-luciferase reporter activity by the BMP9 treatment was apparently low but still statistically significant in the cells expressing the T265P variant (Figure 5B), suggesting that the alteration of T265 to proline might have a milder impact on the interaction between ACVRL1 and SMAD1 via the L45 loop.

### 3.6. Structural Prediction of ACVRL1-BMPR2 Interaction and Influence of Missense Variations in NANDOR and GS Domains

On the other hand, the NANDOR domain (aa 479-489) is located adjacent to the carboxy-terminus of ALK family receptors and plays a central role for the type I–type II receptor complex formation [8,49,50]. A ColabFold prediction indicated the interaction interface between the ACVRL1 kinase domain (aa 195-503) and BMPR2 kinase domain (aa 197-512), in which the R484 residue of ACVRL1 appeared to form salt bridges and close contact with D482 and D485 of BMPR2 (Figure 4A,C). Mutations to this R484 residue would therefore be associated with ACVRL1 molecular dysfunction. Numerous different variations in R484 have been deposited in the ARUP database, such as those resulting in amino acid change to glutamine, glycine, leucine, proline and tryptophan, and Ricard et al. reported functional deficits of R484Q and R484W variants [8]. In this study, the R484L variant showed downregulation of BMP9-induced signal transduction activity while it kept statistical significance (Figure 5A,B). In addition, we found that the R479P variant was completely defective in mediating the BMP9 action (Figure 5A,B). R479 is thoroughly conserved among ACVRL1 proteins in various species (Table 2) as well as other ALK family receptors. We detected the R479P variation in an HHT family in addition to one published case [28] (Table 1), and two other variants, R479L and R479Q, were identified in HHT patients [8,26]. Although ColabFold did not predict that R479 was in direct contact with BMPR2, R479 likely plays an essential role in the ACVRL1 function through a different mechanism.

The other functional motif conserved in ALK family receptors is the GS domain (aa 173-201), which is phosphorylated by BMPR2 upon the BMP ligand binding and acts as a hub for the interaction with SMAD as well as FKBP12 [46]. Unlike the other two functional motifs, ColabFold was not able to show a stable structure of the GS domain when analyzed with BMPR2, SMAD1 and/or FKBP12, preventing us from estimating its importance. Concerning four GS domain variants tested in this paper, the mutations of the T197-equivalent residue (T200) in TGFBR1/ALK5 receptor eliminated TGFβ-induced signal transduction in cell culture experiments [51], but it was not known whether the other three residues were absolutely required for the function of ACVRL1 and other family receptors. Analysis using BRE-luciferase revealed that L193P and T197I variants entirely lacked the signal transduction capacity while D176Y and R200G kept it despite of their pathogenicity predicted by the computational classifiers (Figure 5A,B, Table 2).

### 3.7. Detailed Characterization of Variants without Functional Defects and Responsiveness to Endoglin Co-Expression

**Figure 6:**
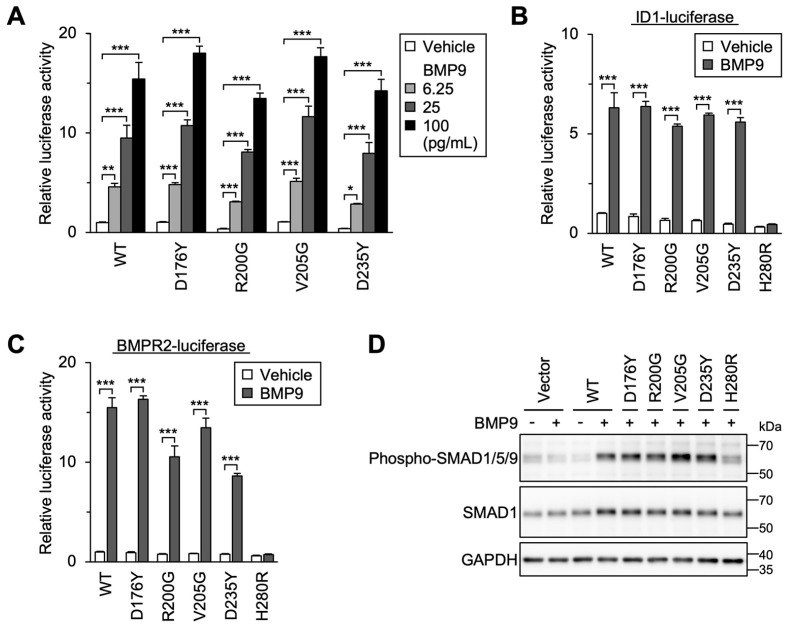
Signal transduction capacity of D176Y, R200G, V205G and D235Y variants. (**A**) D176Y, R200G, V205G and D235Y variants showed BMP9 dose dependency similar to wild-type (WT) ACVRL1 in BRE-luciferase assays. Results are expressed as fold induction over the value obtained for the cells expressing wild-type ACVRL1 with the vehicle treatment. * *p* < 0.05; ** *p* < 0.01; *** *p* < 0.001 (Dunnett’s test). (**B**,**C**) The cells expressing D176Y, R200G, V205G and D235Y variants showed BMP9-induced transcriptional activity in ID1- or BMPR2-luciferase assays. Results are expressed as fold induction over the values obtained for vehicle-treated wild-type ACVRL1. *** *p* < 0.001 (Tukey’s test). (**D**) The cells expressing D176Y, R200G, V205G and D235Y variants showed SMAD1/5/9 phosphorylation in response to the BMP9 treatment, which was comparable to those expressing wild-type ACVRL1. Note that vector-transfected cells did not show significant SMAD1/5/9 phosphorylation. Western blot analysis.

**Figure 7:**
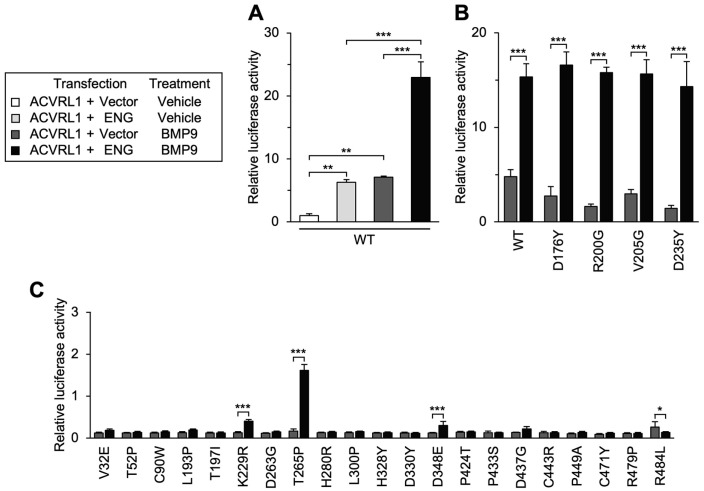
Responsiveness of wild-type ACVRL1 and missense variants to endoglin co-expression. (**A**) Transcription mediated by wild-type (WT) ACVRL1 was increased by the co-expression of endoglin (ENG) in BRE-luciferase assays. Results are expressed as fold induction over the value obtained for the cells with wild-type ACVRL1 expression and vehicle treatment. ** *p* < 0.01; *** *p* < 0.001 (Tukey’s test). (**B**) D176Y, R200G, V205G and D235Y variants also showed significant induction of BMP9-dependent signal transduction when co-expressed with endoglin. BRE-luciferase assays. *** *p* < 0.001 (Tukey’s test). (**C**) Co-expression of endoglin significantly increased the BMP9-induced transcription mediated by K229R, T265P and D348E. In contrast, endoglin significantly decreased the BMP9-induced transcription in the cells expressing R484L. BRE-luciferase assays. In panels B and C, the results are expressed as fold induction over the value obtained for the cells with wild-type ACVRL1 expression and vehicle treatment. The results in panel A serve as a wild-type ACVRL1 control from the same experiment for panel (**C**). * *p* < 0.05; *** *p* < 0.001 (Tukey’s test).

As for D176Y and R200G variants (GS domain) as well as V205G and D235Y variants (kinase domain), we confirmed that they responded to smaller amounts of BMP9 and displayed a normal pattern of dose-dependency in BRE-luciferase assays (Figure 6A). Their function was kept unaffected when different SMAD-dependent luciferase reporters were used, in contrast to the defects of other variants such as H280R (Figure 6B,C), and SMAD1 phosphorylation in response to the BMP9 treatment was equivalent in the cells expressing wild-type ACVRL1 and these four variants (Figure 6D). As previously described [43], the co-expression of endoglin with wild-type ACVRL1 significantly increased the responsiveness to BMP9 (Figure 7A). In this experimental condition, D176Y, R200G, V205G and D235Y variants mediated normal up regulation of BMP9-induced BRE-luciferase activation by the endoglin co-expression (Figure 7B). It is therefore suggested that these ACVRL1 variants can normally mediate SMAD-dependent signaling at least in our experimental systems.

It is worthwhile to mention that the endoglin co-expression significantly increased BMP9-induced BRE-luciferase activity mediated by some functionally defective variants (K229R, T265P and D348E) but decreased that mediated by the R484L variant (Figure 7C), suggesting that ACVRL1 missense variants might have diverse characteristics concerning the endoglin effect.

## 4. Discussion

While previous studies analyzed molecular functions of *ACVRL1* missense variants in cell culture experiments [8,9,10], they did not utilize in silico pathogenicity classifiers to compare the evaluation results. In this study, we performed computational and experimental analyses for the pathogenicity prediction of *ACVRL1* variants that were recently reported for HHT patients (Figure 1, Table 1). We used four in silico pathogenicity classifiers that evaluated the impact of amino acid replacement by the evolutionary conservation of protein sequence and the structural and chemical characteristics of amino acid residues involved in the substitution (Table 2) [7,11,13,41,42]. The extracellular region of ACVRL1 is less conserved across species and compared to other ALK family receptors, which may explain, at least in part, why multiple classifiers miscategorized V32E and T52P as benign, tolerated or neutral. Immunocytochemistry of these proteins clearly showed their pathogenicity due to the impairment of cell surface localization, indicating its usefulness for the variant classification (Figure 2A,B). The analyses using SMAD-dependent luciferase reporters confirmed their functional defects (Figure 2C), providing strong evidence, PS3, in the ACMG criteria classification. Since the predictive accuracy of individual classifiers is relatively low, it is recommended to utilize a combination of in silico tools or an ensemble method, such as M-CAP and REVEL, to obtain improved performance [7,52,53,54]. For example, both V32E and T52P were evaluated to be pathogenic by M-CAP. M-CAP was developed using public database information including patient profiles and results of variant functional analyses to optimize the accuracy of pathogenicity prediction, while it incorporates the evolutionary and structural data from the systems such as SIFT and PolyPhen-2 with its own ways of weighting [53]. On the premise of its accuracy, the usage of clinical and experimental information in in silico classifiers should help better pathogenicity prediction for particular variants when coupled with the evolutional and structural characteristics.

It was unexpected that D176Y, R200G, V205G and D235Y variants maintained high SMAD-dependent signal transduction capacity (Figure 3B, Figure 5B and Figure 6). These amino acid residues are localized in the functional domains of ACVRL1 and are well conserved among species (Figure 1, Table 2). Four pathogenicity classifiers mostly evaluated them to be pathogenic (Table 2), and M-CAP did as well. All those variants have been observed in more than two independent HHT cases. The D176Y variant was reported by a German group [24] and was additionally found in our genetic analysis of a Japanese patient (Table 1). R200G and V205G were identified in the patients from three and two Japanese families, respectively [20]. While D235Y has not been described until this study, we detected it in two probands with a definite HHT diagnosis and confirmed the absence of *ENG* exonic variations by Sanger sequencing (H. Morisaki, unpublished observation). It is therefore suggested that these variations are important as a cause of disease. Although the lack of abnormalities in our experimental systems do not inevitably negate the involvement of SMAD signaling, it will be necessary to analyze other mechanisms of variant protein dysfunction including that related to non-SMAD-dependent protein kinase signaling [55,56,57]. Furthermore, it is important to note that the exonic variations may also lead to splicing defects [9,58]. An in silico analysis of 25 variants using MaxEntScan [59] suggested that the c.703G>T (D235Y) substitution could create an aberrant 5’-splice site in the middle of *ACVRL1* exon 6. Future studies may well have to test such a possibility by performing minigene splicing experiments as well as characterizing the *ACVRL1* mRNA splicing in patient tissues and cells.

Additionally, V32E, T265P, D437G and R484L variants kept low but statistically significant levels of SMAD-dependent signal transduction capacity while it was markedly down regulated compared to that of wild-type protein (Figure 2C, Figure 3B and Figure 5B). The patients carrying one of these variations satisfied the definite Curaçao criteria [19,24,35,36] (Table 1), and we assume that these variations are pathogenic despite such residual activity. Remaining signaling activity in the variants does not necessarily corelate with the positive effects by the endoglin co-expression (Figure 7). Among these four variants, only T265P possessed the endoglin responsiveness and R484L even showed decreased SMAD-dependent signaling in the condition with endoglin co-expression. In contrast, K229R and D348E, which did not mediate BMP9-induced SMAD signal activation without the endoglin co-expression (Figure 3B), displayed a significant response when co-expressed with endoglin. On the assumption that at least particular variant proteins may remain in patient cells without degradation, understanding diverse properties of different variants may help clarify fine pathophysiology in each patient.

ACVRL1 forms the receptor complex with BMPR2 and endoglin and also interacts with the ligands, functional modulators and effector proteins [2]. Although the monomer structure of ACVRL1 intracellular kinase domain was predicted by the homology modeling and determined by crystallography [8,60,61], how ACVRL1 interacts with its partners has not been analyzed. The ColabFold-based structural prediction confirmed the importance of the L45 loop and NANDOR domain for the association of ACVRL1 with SMAD1 and BMPR2, respectively (Figure 4) [49,62]. Missense variants of these motifs showed apparent deficiency in SMAD-dependent signaling (Figure 5B), which suggested the compromised protein interaction as a mechanism of abnormalities in the patients with those variations. In this study, however, we were not able to detect significant changes of protein complex structures when variant ACVRL1 proteins were used for the ColabFold analyses. Current in silico pathogenicity classifiers do not provide insight into the mechanisms of variant protein dysfunction, and so, it will be highly beneficial if we can utilize the computational prediction to estimate the impact of variations upon the single chain protein structure as well as the protein complex formation.

The present study evaluated the influence of *ACVRL1* missense variations in its functional domains and motifs using computational pathogenicity classification, three-dimensional structure prediction and experimental studies for cellular localization and signal transduction. The results in different analyses were sometimes inconsistent with each other, suggesting that a combinatory approach was all the more important to precisely estimate the pathogenicity of ACVRL1 missense variants found in HHT patients.

## Figures and Tables

**Table 2:** Sequence conservation of ACVRL1 amino acid residues and computational pathogenicity prediction of missense variants.

Variant	Homology	Computational Pathogenicity Classifier			
	MCLXZ	SIFT	PolyPhen-2	PROVEAN	PANTHER
V32E	VLVWL	Tolerated	Benign	Neutral	Poss Dmgg
T52P	TFFYF	Tolerated	Prob Dmgg	Neutral	Poss Dmgg
C90W	CCCCC	Deleterious	Prob Dmgg	Deleterious	Prob Dmgg
D176Y	DDNED	Deleterious	Prob Dmgg	Deleterious	Poss Dmgg
L193P	LLLLL	Deleterious	Prob Dmgg	Deleterious	Prob Dmgg
T197I	TTTTT	Deleterious	Prob Dmgg	Deleterious	Prob Dmgg
R200G	RRRRR	Deleterious	Prob Dmgg	Deleterious	Prob Dmgg
V205G	VVVVV	Deleterious	Prob Dmgg	Deleterious	Poss Dmgg
K229R	KKKKK	Deleterious	Prob Dmgg	Deleterious	Poss Dmgg
D235Y	DDDDD	Deleterious	Prob Dmgg	Deleterious	Poss Dmgg
D263G	DDDDD	Deleterious	Prob Dmgg	Deleterious	Prob Dmgg
T265P	TTTTT	Tolerated	Prob Dmgg	Deleterious	Prob Dmgg
H280R	HHHHH	Deleterious	Prob Dmgg	Deleterious	Prob Dmgg
L300P	LLLLL	Deleterious	Prob Dmgg	Deleterious	Prob Dmgg
H328Y	HHHHH	Deleterious	Prob Dmgg	Deleterious	Prob Dmgg
D330Y	DDDDD	Deleterious	Prob Dmgg	Deleterious	Prob Dmgg
D348E	DDDDD	Deleterious	Prob Dmgg	Deleterious	Prob Dmgg
P424T	PPPPP	Deleterious	Prob Dmgg	Deleterious	Prob Dmgg
P433S	PPPPP	Deleterious	Prob Dmgg	Deleterious	Prob Dmgg
D437G	DDDDE	Deleterious	Prob Dmgg	Deleterious	Poss Dmgg
C443R	CCCCC	Deleterious	Prob Dmgg	Deleterious	Prob Dmgg
P449A	PPPPP	Deleterious	Prob Dmgg	Deleterious	Prob Dmgg
C471Y	CCCCC	Deleterious	Prob Dmgg	Deleterious	Prob Dmgg
R479P	RRRRR	Deleterious	Prob Dmgg	Deleterious	Prob Dmgg
R484L	RRRRR	Deleterious	Prob Dmgg	Deleterious	Prob Dmgg

Conservation of ACVRL1 residues involved in the variations is shown by listing the amino acids in five vertebrate species (M, mouse; C, chicken; L, lizard; X, xenopus; Z, zebrafish). Poss Dmgg, Possibly damaging; Prob Dmgg, Probably damaging.

## Data Availability

Information concerning the experimental data, materials and methods presented in this study are available on request from the corresponding author. The clinical information is not publicly available due to the ethical reason.

## Supplementary Materials

The following supporting information can be downloaded at: https://www.mdpi.com/article/10.3390/jcm12155002/s1, Characteristics of unpublished HHT cases.

## References

[1] Shovlin C.L., Guttmacher A.E., Buscarini E., Faughnan M.E., Hyland R.H., Westermann C.J., Kjeldsen A.D., Plauchu H. (2000). Diagnostic criteria for hereditary hemorrhagic telangiectasia (Rendu-Osler-Weber syndrome). Am. J. Med. Genet..

[2] Bernabeu C., Bayrak-Toydemir P., McDonald J., Letarte M. (2020). Potential Second-Hits in Hereditary Hemorrhagic Telangiectasia. J. Clin. Med..

[3] Robert F., Desroches-Castan A., Bailly S., Dupuis-Girod S., Feige J.J. (2020). Future treatments for hereditary hemorrhagic telangiectasia. Orphanet J. Rare Dis..

[4] Oh S.P., Seki T., Goss K.A., Imamura T., Yi Y., Donahoe P.K., Li L., Miyazono K., ten Dijke P., Kim S. (2000). Activin receptor-like kinase 1 modulates transforming growth factor-beta 1 signaling in the regulation of angiogenesis. Proc. Natl. Acad. Sci. USA.

[5] Urness L.D., Sorensen L.K., Li D.Y. (2000). Arteriovenous malformations in mice lacking activin receptor-like kinase-1. Nat. Genet..

[6] Roman B.L., Pham V.N., Lawson N.D., Kulik M., Childs S., Lekven A.C., Garrity D.M., Moon R.T., Fishman M.C., Lechleider R.J. (2002). Disruption of acvrl1 increases endothelial cell number in zebrafish cranial vessels. Development.

[7] Richards S., Aziz N., Bale S., Bick D., Das S., Gastier-Foster J., Grody W.W., Hegde M., Lyon E., Spector E. (2015). Standards and guidelines for the interpretation of sequence variants: A joint consensus recommendation of the American College of Medical Genetics and Genomics and the Association for Molecular Pathology. Genet. Med..

[8] Ricard N., Bidart M., Mallet C., Lesca G., Giraud S., Prudent R., Feige J.J., Bailly S. (2010). Functional analysis of the BMP9 response of ALK1 mutants from HHT2 patients: A diagnostic tool for novel ACVRL1 mutations. Blood.

[9] Alaa El Din F., Patri S., Thoreau V., Rodriguez-Ballesteros M., Hamade E., Bailly S., Gilbert-Dussardier B., Abou Merhi R., Kitzis A. (2015). Functional and splicing defect analysis of 23 ACVRL1 mutations in a cohort of patients affected by Hereditary Hemorrhagic Telangiectasia. PLoS ONE.

[10] Hume A.N., John A., Akawi N.A., Al-Awadhi A.M., Al-Suwaidi S.S., Al-Gazali L., Ali B.R. (2013). Retention in the endoplasmic reticulum is the underlying mechanism of some hereditary haemorrhagic telangiectasia type 2 ALK1 missense mutations. Mol. Cell Biochem..

[11] Ng P.C., Henikoff S. (2001). Predicting deleterious amino acid substitutions. Genome Res..

[12] Choi Y., Chan A.P. (2015). PROVEAN web server: A tool to predict the functional effect of amino acid substitutions and indels. Bioinformatics.

[13] Adzhubei I.A., Schmidt S., Peshkin L., Ramensky V.E., Gerasimova A., Bork P., Kondrashov A.S., Sunyaev S.R. (2010). A method and server for predicting damaging missense mutations. Nat. Methods.

[14] Tang H., Thomas P.D. (2016). PANTHER-PSEP: Predicting disease-causing genetic variants using position-specific evolutionary preservation. Bioinformatics.

[15] Korchynskyi O., ten Dijke P. (2002). Identification and Functional Characterization of Distinct Critically Important Bone Morphogenetic Protein-specific Response Elements in the Id1 Promoter. J. Biol. Chem..

[16] Morikawa M., Koinuma D., Tsutsumi S., Vasilaki E., Kanki Y., Heldin C.H., Aburatani H., Miyazono K. (2011). ChIP-seq reveals cell type-specific binding patterns of BMP-specific Smads and a novel binding motif. Nucleic Acids Res..

[17] van Meeteren L.A., Thorikay M., Bergqvist S., Pardali E., Stampino C.G., Hu-Lowe D., Goumans M.J., ten Dijke P. (2012). Anti-human activin receptor-like kinase 1 (ALK1) antibody attenuates bone morphogenetic protein 9 (BMP9)-induced ALK1 signaling and interferes with endothelial cell sprouting. J. Biol. Chem..

[18] Mirdita M., Schütze K., Moriwaki Y., Heo L., Ovchinnikov S., Steinegger M. (2022). ColabFold: Making protein folding accessible to all. Nat. Methods.

[19] Komiyama M., Ishiguro T., Yamada O., Morisaki H., Morisaki T. (2014). Hereditary hemorrhagic telangiectasia in Japanese patients. J. Hum. Genet..

[20] Kitayama K., Ishiguro T., Komiyama M., Morisaki T., Morisaki H., Minase G., Hamanaka K., Miyatake S., Matsumoto N., Kato M. (2021). Mutational and clinical spectrum of Japanese patients with hereditary hemorrhagic telangiectasia. BMC Med. Genom..

[21] Nishimoto Y., Morisaki H., Yamada O., Ichinose Y., Suzuki N. (2014). Japanese case of hereditary hemorrhagic telangiectasia type 2 with a novel mutation, c.154A>C (p.Thr52Pro), in the ALK1/ACVRL1 gene. Neurol. Clin. Neurosci..

[22] Iwasa T., Yamada O., Ohuchi H., Shiraishi I., Morisaki H., Morisaki T., Kurosaki K. (2020). A Case of Spontaneously Improved Heritable Pulmonary Arterial Hypertension Diagnosed as Severe Primary Pulmonary Hypertension in Childhood. J. Pediatr. Cardiol. Card. Surg..

[23] Landrum M.J., Lee J.M., Riley G.R., Jang W., Rubinstein W.S., Church D.M., Maglott D.R. (2014). ClinVar: Public archive of relationships among sequence variation and human phenotype. Nucleic Acids Res..

[24] Argyriou L., Twelkemeyer S., Panchulidze I., Wehner L.E., Teske U., Engel W., Nayernia K. (2006). Novel mutations in the ENG and ACVRL1 genes causing hereditary hemorrhagic teleangiectasia. Int. J. Mol. Med..

[25] Bossler A.D., Richards J., George C., Godmilow L., Ganguly A. (2006). Novel mutations in ENG and ACVRL1 identified in a series of 200 individuals undergoing clinical genetic testing for hereditary hemorrhagic telangiectasia (HHT): Correlation of genotype with phenotype. Hum. Mutat..

[26] Lesca G., Plauchu H., Coulet F., Lefebvre S., Plessis G., Odent S., Riviere S., Leheup B., Goizet C., Carette M.F. (2004). Molecular screening of ALK1/ACVRL1 and ENG genes in hereditary hemorrhagic telangiectasia in France. Hum. Mutat..

[27] Heimdal K., Dalhus B., Rødningen O.K., Kroken M., Eiklid K., Dheyauldeen S., Røysland T., Andersen R., Kulseth M.A. (2016). Mutation analysis in Norwegian families with hereditary hemorrhagic telangiectasia: Founder mutations in ACVRL1. Clin. Genet..

[28] Richards-Yutz J., Grant K., Chao E.C., Walther S.E., Ganguly A. (2010). Update on molecular diagnosis of hereditary hemorrhagic telangiectasia. Hum. Genet..

[29] Gedge F., McDonald J., Phansalkar A., Chou L.S., Calderon F., Mao R., Lyon E., Bayrak-Toydemir P. (2007). Clinical and analytical sensitivities in hereditary hemorrhagic telangiectasia testing and a report of de novo mutations. J. Mol. Diagn..

[30] Nishida T., Faughnan M.E., Krings T., Chakinala M., Gossage J.R., Young W.L., Kim H., Pourmohamad T., Henderson K.J., Schrum S.D. (2012). Brain arteriovenous malformations associated with hereditary hemorrhagic telangiectasia: Gene-phenotype correlations. Am. J. Med. Genet. A.

[31] Olivieri C., Pagella F., Semino L., Lanzarini L., Valacca C., Pilotto A., Corno S., Scappaticci S., Manfredi G., Buscarini E. (2007). Analysis of ENG and ACVRL1 genes in 137 HHT Italian families identifies 76 different mutations (24 novel). Comparison with other European studies. J. Hum. Genet..

[32] Fontalba A., Fernandez-L A., García-Alegria E., Albiñana V., Garrido-Martin E.M., Blanco F.J., Zarrabeitia R., Perez-Molino A., Bernabeu-Herrero M.E., Ojeda M.-L. (2008). Mutation study of Spanish patients with Hereditary Hemorrhagic Telangiectasia. BMC Med. Genet..

[33] Argyriou L., Pfitzmann R., Wehner L.E., Twelkemeyer S., Neuhaus P., Nayernia K., Engel W. (2005). ALK-1 mutations in liver transplanted patients with hereditary hemorrhagic telangiectasia. Liver Transpl..

[34] Letteboer T.G., Zewald R.A., Kamping E.J., de Haas G., Mager J.J., Snijder R.J., Lindhout D., Hennekam F.A., Westermann C.J., Ploos van Amstel J.K. (2005). Hereditary hemorrhagic telangiectasia: ENG and ALK-1 mutations in Dutch patients. Hum. Genet..

[35] McDonald J., Damjanovich K., Millson A., Wooderchak W., Chibuk J.M., Stevenson D.A., Gedge F., Bayrak-Toydemir P. (2011). Molecular diagnosis in hereditary hemorrhagic telangiectasia: Findings in a series tested simultaneously by sequencing and deletion/duplication analysis. Clin. Genet..

[36] Brakensiek K., Frye-Boukhriss H., Malzer M., Abramowicz M., Bahr M.J., von Beckerath N., Bergmann C., Caselitz M., Holinski-Feder E., Muschke P. (2008). Detection of a significant association between mutations in the ACVRL1 gene and hepatic involvement in German patients with hereditary haemorrhagic telangiectasia. Clin. Genet..

[37] Machado R.D., Southgate L., Eichstaedt C.A., Aldred M.A., Austin E.D., Best D.H., Chung W.K., Benjamin N., Elliott C.G., Eyries M. (2015). Pulmonary Arterial Hypertension: A Current Perspective on Established and Emerging Molecular Genetic Defects. Hum. Mutat..

[38] Berg J.N., Gallione C.J., Stenzel T.T., Johnson D.W., Allen W.P., Schwartz C.E., Jackson C.E., Porteous M.E.M., Marchuk D.A. (1997). The Activin Receptor-Like Kinase 1 Gene: Genomic Structure and Mutations in Hereditary Hemorrhagic Telangiectasia Type 2. Am. J. Hum. Genet..

[39] Karczewski K.J., Francioli L.C., Tiao G., Cummings B.B., Alföldi J., Wang Q., Collins R.L., Laricchia K.M., Ganna A., Birnbaum D.P. (2020). The mutational constraint spectrum quantified from variation in 141,456 humans. Nature.

[40] Tadaka S., Hishinuma E., Komaki S., Motoike I.N., Kawashima J., Saigusa D., Inoue J., Takayama J., Okamura Y., Aoki Y. (2020). jMorp updates in 2020: Large enhancement of multi-omics data resources on the general Japanese population. Nucleic Acids Res..

[41] Choi Y., Sims G.E., Murphy S., Miller J.R., Chan A.P. (2012). Predicting the Functional Effect of Amino Acid Substitutions and Indels. PLoS ONE.

[42] Thomas P.D., Campbell M.J., Kejariwal A., Mi H., Karlak B., Daverman R., Diemer K., Muruganujan A., Narechania A. (2003). PANTHER: A library of protein families and subfamilies indexed by function. Genome Res..

[43] Mallet C., Lamribet K., Giraud S., Dupuis-Girod S., Feige J.J., Bailly S., Tillet E. (2015). Functional analysis of endoglin mutations from hereditary hemorrhagic telangiectasia type 1 patients reveals different mechanisms for endoglin loss of function. Hum. Mol. Genet..

[44] González-Núñez M., Muñoz-Félix J.M., López-Novoa J.M. (2013). The ALK-1/Smad1 pathway in cardiovascular physiopathology. A new target for therapy?. Biochim. Biophys. Acta (BBA) Mol. Basis Dis..

[45] Chaikuad A., Alfano I., Kerr G., Sanvitale C.E., Boergermann J.H., Triffitt J.T., von Delft F., Knapp S., Knaus P., Bullock A.N. (2012). Structure of the bone morphogenetic protein receptor ALK2 and implications for fibrodysplasia ossificans progressiva. J. Biol. Chem..

[46] Martinez-Hackert E., Sundan A., Holien T. (2021). Receptor binding competition: A paradigm for regulating TGF-β family action. Cytokine Growth Factor Rev..

[47] Wu G., Chen Y.G., Ozdamar B., Gyuricza C.A., Chong P.A., Wrana J.L., Massagué J., Shi Y. (2000). Structural basis of Smad2 recognition by the Smad anchor for receptor activation. Science.

[48] Wu J.-W., Hu M., Chai J., Seoane J., Huse M., Li C., Rigotti D.J., Kyin S., Muir T.W., Fairman R. (2001). Crystal Structure of a Phosphorylated Smad2: Recognition of Phosphoserine by the MH2 Domain and Insights on Smad Function in TGF-β Signaling. Mol. Cell.

[49] Agnew C., Ayaz P., Kashima R., Loving H.S., Ghatpande P., Kung J.E., Underbakke E.S., Shan Y., Shaw D.E., Hata A. (2021). Structural basis for ALK2/BMPR2 receptor complex signaling through kinase domain oligomerization. Nat. Commun..

[50] Garamszegi N., Doré J.J.E., Penheiter S.G., Edens M., Yao D., Leof E.B. (2001). Transforming Growth Factor β Receptor Signaling and Endocytosis Are Linked through a COOH Terminal Activation Motif in the Type I Receptor. Mol. Biol. Cell.

[51] Doré J.J., Yao D., Edens M., Garamszegi N., Sholl E.L., Leof E.B. (2001). Mechanisms of transforming growth factor-beta receptor endocytosis and intracellular sorting differ between fibroblasts and epithelial cells. Mol. Biol. Cell.

[52] Thusberg J., Olatubosun A., Vihinen M. (2011). Performance of mutation pathogenicity prediction methods on missense variants. Hum. Mutat..

[53] Jagadeesh K.A., Wenger A.M., Berger M.J., Guturu H., Stenson P.D., Cooper D.N., Bernstein J.A., Bejerano G. (2016). M-CAP eliminates a majority of variants of uncertain significance in clinical exomes at high sensitivity. Nat. Genet..

[54] Ioannidis N.M., Rothstein J.H., Pejaver V., Middha S., McDonnell S.K., Baheti S., Musolf A., Li Q., Holzinger E., Karyadi D. (2016). REVEL: An Ensemble Method for Predicting the Pathogenicity of Rare Missense Variants. Am. J. Hum. Genet..

[55] Qu X., Liu Y., Cao D., Chen J., Liu Z., Ji H., Chen Y., Zhang W., Zhu P., Xiao D. (2019). BMP10 preserves cardiac function through its dual activation of SMAD-mediated and STAT3-mediated pathways. J. Biol. Chem..

[56] Derynck R., Budi E.H. (2019). Specificity, versatility, and control of TGF-beta family signaling. Sci. Signal..

[57] Shim J.H., Greenblatt M.B., Xie M., Schneider M.D., Zou W., Zhai B., Gygi S., Glimcher L.H. (2009). TAK1 is an essential regulator of BMP signalling in cartilage. EMBO J..

[58] Soemedi R., Cygan K.J., Rhine C.L., Wang J., Bulacan C., Yang J., Bayrak-Toydemir P., McDonald J., Fairbrother W.G. (2017). Pathogenic variants that alter protein code often disrupt splicing. Nat. Genet..

[59] Yeo G., Burge C.B. (2004). Maximum entropy modeling of short sequence motifs with applications to RNA splicing signals. J. Comput. Biol..

[60] Abdalla S.A., Cymerman U., Johnson R.M., Deber C.M., Letarte M. (2003). Disease-associated mutations in conserved residues of ALK-1 kinase domain. Eur. J. Hum. Genet..

[61] Kerr G., Sheldon H., Chaikuad A., Alfano I., von Delft F., Bullock A.N., Harris A.L. (2015). A small molecule targeting ALK1 prevents Notch cooperativity and inhibits functional angiogenesis. Angiogenesis.

[62] Chen Y.G., Massagué J. (1999). Smad1 recognition and activation by the ALK1 group of transforming growth factor-beta family receptors. J. Biol. Chem..

