# Supplementary material for: Computational and Experimental Analyses for Pathogenicity Prediction of ACVRL1 Missense Variants in Hereditary Hemorrhagic Telangiectasia
Source: J Clin Med. 2023 Jul 29;12(15):5002. doi: 10.3390/jcm12155002 (PMC10419700; doi:10.3390/jcm12155002)
Supplement: Supplementary file 1 [file jcm-12-05002-s001.zip › jcm-2475453-supplementary.pdf]

Supplementary Information

Characteristics of unpublished HHT cases

Clinical profiles of unpublished HHT patients with an *ACVRL1* missense variation were summarized in Table 1. The information of the patients with a c.703G>T (D235Y) variation was described in the Results section.

The patient with a c.526G>T (D176Y) variation was a 67-year-old man who had experienced recurrent epistaxis since he was approximately 60 years old. Telangiectasia on the nasal mucosa was found when the laser treatment was performed. No vascular malformation in the brain, lung and liver was pointed out. His uncle and daughter also experienced recurrent epistaxis, while they were not evaluated for the HHT diagnosis.

The patient with a c.1270C>A (P424T) variation was a 61-year-old woman with recurrent nosebleed for about 30 years. Laser surgery for prolonged nasal bleeding was performed at 59 years of age. She also experienced bleeding from the tongue at the age of 52. She had telangiectasias on the face and lip, and enhanced computed tomography revealed hepatic arteriovenous shunts, but no vascular abnormality was detected in the brain and lung.

The c.1310A>G (D437G) variation was found in an 81-year-old woman with pulmonary and hepatic vascular malformations. Her son with the same variation experienced recurrent epistaxis and had multiple vascular malformations in the lung, while her daughter without the variation showed no HHT-like symptoms.

The c.1436G>C (R479P) variation was identified in a 28-year-old-woman. She presented recurrent epistaxis and telangiectasias on the face and suffered from severe pulmonary arterial hypertension. She had identical twin brothers that carried an R479 variation. One of them experienced recurrent epistaxis, but the other was asymptomatic. Her asymptomatic father was negative for the R479P variation.

The patient with a c.1451G>T (R484L) variation was a 49-year-old woman. She was diagnosed as pulmonary arterial hypertension at the age of 33, but the anticoagulation therapy was terminated due to recurrent epistaxis. Abdominal ultrasound sonography revealed multiple arteriovenous shunts in the liver. Her father, grandfather, aunt, sister and niece also experienced recurrent nosebleed, while their clinical information was not known in detail.

These five variants were previously reported only in one case [24,28,35,36,38], and the patients described here were second unrelated cases.
